# Metabolic subtyping reveals PDIK1L as a dual-functional regulator of progression and PARP inhibitor sensitivity in prostate cancer

**DOI:** 10.3389/fcell.2025.1674844

**Published:** 2025-11-04

**Authors:** Zhongyuan Wang, Qintao Ge, Anwaier Aihetaimujiang, Ji Zhang, Jiahe Lu, Jianfeng Yang, Yonghao Chen, Bin Qin, Hailiang Zhang, Wen-Hao Xu, Dingwei Ye

**Affiliations:** ^1^ Department of Oncology, Shanghai Medical College, Shanghai, China; ^2^ Department of Urology, Fudan University Shanghai Cancer Center, Shanghai, China; ^3^ Fudan University, Shanghai, China; ^4^ Department of Nursing, Fudan University Shanghai Cancer Center, Shanghai, China; ^5^ Department of Urology, Longhua Hospital, Shanghai University of Traditional Chinese Medicine, Shanghai, China; ^6^ Sichuan University West China Medical Center, Chengdu, Sichuan, China; ^7^ Orthopedics department, Wuxi Hospital of Traditional Chinese Medicine, Wuxi, China

**Keywords:** prostate cancer, metabolic subtyping, PARP inhibitor, genomic instability, durg resistance

## Abstract

**Background:**

Prostate cancer demonstrates significant metabolic heterogeneity, but its role in therapeutic resistance and disease progression remains unclear. This study investigates the clinical implications of metabolic diversity and identifies potential biomarkers for precision oncology.

**Methods:**

Multi-omics analyses of TCGA-PRAD and meta-cohorts classified tumors into three metabolic subtypes (C1, C2, C3). Functional studies utilized prostate cancer cell lines with genetic modulation of PDIK1L. Proliferation assays, protein expression analysis, and drug sensitivity evaluations were systematically performed.

**Results:**

Metabolic subtyping delineated distinct molecular and clinical profiles. The C2 subtype demonstrated elevated genomic instability and heightened sensitivity to PARP inhibitors, characterized by enrichment of glycogen metabolism and TP53-driven oncogenic pathways. Integrative multi-omics and random survival forest analysis prioritized PDIK1L as a C2-specific biomarker, where its overexpression accelerated tumor proliferation and rewired metabolic programs to confer resistance to PARP inhibitors. Conversely, PDIK1L knockdown suppressed proliferation and sensitized cells to therapy, underscoring its role as a dual-functional regulator. Mechanistically, PDIK1L interacted with DNA repair and metabolic adaptation pathways, creating a permissive environment for therapeutic resistance. Combinatorial therapy with Enzalutamide and PARP inhibitors effectively reversed PDIK1L-mediated resistance, restoring drug sensitivity across preclinical models. Independent validation in multi-institutional cohorts confirmed the robustness of metabolic subtyping and PDIK1L’s prognostic value in predicting survival and treatment outcomes.

**Discussion:**

Metabolic stratification reveals the C2 subtype as a high-risk prostate cancer group with unique therapeutic vulnerabilities. PDIK1L emerges as a dual-functional biomarker driving tumor progression and modulating treatment efficacy, offering a novel target for precision therapeutic strategies.

## Introduction

Prostate cancer is the second most common malignancy among men globally, with the number of new cases projected to rise from 1.4 million in 2020 to 2.9 million by 2040 (Bray et al., 2024; James et al., 2024). This disease is characterized by significant clinical and molecular heterogeneity (Tang et al., 2022; Han et al., 2022; Dai et al., 2024). While advancements in androgen deprivation therapy, precision-targeted therapy, and radiotherapy have improved patient outcomes, treatment resistance and disease progression remain critical challenges (Li et al., 2023; He et al., 2022). Emerging evidence highlights metabolic reprogramming as a hallmark of cancer, yet the role of metabolic diversity in prostate cancer has not been fully explored (Ward and Thompson, 2012; Allison et al., 2017). Tumor cells exhibit dynamic changes in nutrient utilization, including glycogen synthesis, glycolysis, glutaminolysis, and lipid metabolism, to promote proliferation, metastasis, and adapt to microenvironment stress (Huang et al., 2025; Perez et al., 2024; Zhou et al., 2024). This metabolic plasticity drives tumor evolution and contributes to inter- and intra-tumor heterogeneity, complicating therapeutic stratification. Importantly, metabolic heterogeneity may underpin differential responses to therapies, including radiotherapy, targeted therapy, and androgen deprivation therapy (Venkatraman et al., 2024; Yao et al., 2024; Cardoso et al., 2021). By analyzing the metabolic profiles of prostate cancer subtypes, researchers can identify actionable targets that align with clinical phenotypes, providing a framework for refining prognostic models and personalizing therapeutic interventions.

The metabolic diversity in cancer is rooted in genetic, epigenetic, and microenvironmental factors that shape tumor behavior (You et al., 2023). For example, mutations in PTEN, TP53, and MYC drive distinct metabolic programs, while hypoxia and nutrient scarcity within the tumor microenvironment (TME) compel metabolic adaptations (Venkatraman et al., 2024; Morris et al., 2019; Chen et al., 2018). Stromal cells, immune infiltration, and extracellular matrix components further modulate these metabolic interactions, creating a milieu conducive to therapeutic resistance (Lyu et al., 2025). For instance, lipid-rich niches may protect cancer stem cells from oxidative stress (Snaebjornsson et al., 2020), whereas lactate secretion by glycolytic tumors can suppress anti-tumor immunity (Chaudagar et al., 2023). Moreover, androgen receptor signaling orchestrates metabolic pathways such as fatty acid synthesis and mitochondrial respiration, linking hormone dependency with metabolic dependency (Zadra et al., 2019; Han et al., 2018). These intricate interactions suggest that metabolic heterogeneity not only plays a bystander role but also acts as a determinant of clinical trajectories. Therefore, integrating metabolomics, transcriptomics, and genomics data may unveil biomarkers for risk stratification and identify metabolic nodes susceptible to pharmacological inhibition.

Translating the metabolic diversity of prostate cancer into actionable therapeutic strategies could revolutionize clinical outcomes. This research analyzes how metabolic heterogeneity impacts treatment resistance and progression in prostate cancer, highlighting the necessity of integrating metabolic subtypes with genomic and clinical data. This approach refines risk stratification and uncovers context-specific vulnerabilities, linking metabolic heterogeneity with precision oncology. Consequently, it aims to guide improvements in patient survival and quality of life.

## Methods and materials

### Row data collection and processing

We integrated three independent cohorts with complete expression profiles and clinical follow-up data, including the TCGA PRAD cohort (n = 495, from the GDC platform: https://portal.gdc.cancer.gov/), the MSKCC Prostate Cancer Genomics Project (MSKCC, n = 140, https://cbio.mskcc.org/), and the GSE70770 cohort (n = 203).The expression matrices of all samples were uniformly annotated according to their respective database platforms and converted to TPM (transcripts per million) format. We used recurrence-free survival (RFS) as the primary endpoint to evaluate the clinical outcomes of PCa patients. Batch effects were removed by ‘sva’ package (Leek et al., 2012) (Supplementary Figure S1A).

### Non-negative matrix factorization (NMF) clustering and nearest template prediction (NTP)

Drawing on earlier research, we created a compilation of 2,752 genes associated with metabolism to use as input for NMF clustering (Possemato et al., 2011). Prior to executing the NMF, we performed a screening procedure that entailed the elimination of candidate genes exhibiting low median absolute deviation (MAD) values (MAD ≤0.5) among PCa patients. This procedure encompassed Cox regression analysis, the identification of shared genes from three different cohorts, and the ultimate selection of genes that demonstrated high variability (MAD >0.5) along with notable prognostic significance (*P* < 0.05) for clustering samples. The ideal number of clusters was established by pinpointing the k value at which the co-phenotypic correlation coefficient started to decrease (Gaujoux and Seoighe, 2010; Brunet et al., 2004). We confirmed the assignment of subtypes utilizing a methodology based on t-distributed stochastic neighbor embedding (t-SNE) with the mRNA expression data from the previously mentioned metabolic genes.

In order to predict patient subtypes across different cohorts, we began by identifying particular genes that showed notable differences in expression when comparing all possible pairs among the three subtypes (Supplementary Table S1). The threshold for these differences was established with an adjusted *P*-value. We then chose the top 30 genes for each subtype to create the feature set for subtype prediction. Utilizing this feature set, we applied the NTP algorithm to reassign subtypes within the validation cohort.

### Gene set variation analysis

To evaluate metabolic function and the progression of disease quantitatively, we applied the Gene Set Variation Analysis (GSVA) approach to carry out pathway enrichment scoring on the samples (Hoshida et al., 2009). The gsva package (Hanzelmann et al., 2013) was utilized in the analysis of the standardized transcriptomic data. Following this, the limma package was used to conduct differential analysis on the GSVA scores of 115 metabolic labels, applying criteria |log2FC|>0.2 and a Benjamini–Hochberg adjusted *P*-value <0.05.

### Mutation analysis

To comprehensively assess the genomic instability and immune-related features of PCa patients within various molecular subtypes from TCGA, we developed an all-encompassing analytical pipeline that includes mutation profiles, tumor mutation burden (TMB) analysis, and copy number variation (CNV) evaluation. MAF format of mutation data was downloaded from FireBrowse (http://firebrowse.org/), maftools and ComplexHeatmap package was employed to generate an OncoPrint visualization, showcasing the types of mutations and their distribution across samples from each subtype. Following that, we used maftools to compute TMB. To investigate the co-mutation relationships of key driver genes among different subtypes of PCa, we utilized the somaticInteractions () function from the maftools package, focusing on 24 high-frequency mutated genes. This function was employed to calculate the co-occurrence and mutual exclusivity of gene pairs within the three subtypes (C1-C3). Furthermore, to systematically assess the extent of functional abnormalities in key cancer signaling pathways across various subtypes, we integrated common tumor-associated pathways (including RTK-RAS, WNT, NOTCH, PI3K, TP53, MYC, etc.) and employed the OncogenicPathways () function to identify the impact of each pathway within the samples.

### Evaluation of tumor immune microenvironment, immunomodulator compilation and immunotherapy efficacy

To systematically evaluate the immune characteristics of different subtypes of PCa, we conducted an in-depth analysis of mRNA expression profiles related to immune regulation, integrating multi-omics alterations, including methylation modifications, copy number amplifications, and gene deletions. This analysis builds upon and extends the research framework established in previous literature (Thorsson et al., 2018). Additionally, starting from 21 known immune-related gene sets, we scored and visualized the immune pathway activities of each subtype sample using the GSVA algorithm integrated within the MOVICS package (Meng et al., 2021; Lu et al., 2021). To further investigate the mechanisms of tumor immune evasion and the potential responsiveness to immunotherapy, we downloaded the TIDE scores of PCa samples from the TIDE platform (https://tide.dfci.harvard.edu/) to quantify the extent of immune dysfunction and immune exclusion, thereby providing a basis for clinical decision-making in immunotherapy.

### Spatial transcriptomics data processing and module scoring analysis

The spatial transcriptomics data were acquired using the 10x Genomics platform (https://www.10xgenomics.com/). The raw data, which includes the gene expression matrix and tissue images, were downloaded from the official 10x Genomics website and imported to create a Seurat object using the Load10X_Spatial () function from the Seurat package. Based on the group-specific differentially expressed genes and functional modules defined earlier, we employed the AddModuleScore () function for the spatial visualization of different groups. The TLS (Tertiary Lymphoid Structure) genes can be sourced from our previous publication (Xu et al., 2023; Xu et al., 2020). The scoring results were visualized spatially using the SpatialFeaturePlot () function, while the expression distribution differences for each score in TLS and non-TLS regions were depicted using the VlnPlot () function.

### Random forest model

In order to pinpoint feature genes that may have diagnostic or subtyping relevance, we performed an evaluation of feature significance utilizing the Random Forest model. The classification model was developed using the R package randomForest, with a total of 2000 decision trees configured. To assess the stability of the model, we generated error curves. From the MeanDecreaseAccuracy and MeanDecreaseGini indices, we identified the top 20 crucial genes that played a significant role in classification for further analysis and validation.

### Cell lines and cell culture

C4-2 and PC-3 were obtained from the Shanghai Cell Bank of Chinese Academy of Sciences (Shanghai, China). Both cell lines were maintained in RPMI-1640 medium (Gibco) supplemented with 10% fetal bovine serum (FBS; Gibco) and 1% penicillin-streptomycin (Sigma) at 37 °C in a humidified atmosphere containing 5% CO_2_. Culture medium was refreshed every 2–3 days, and cells were subcultured using 0.25% trypsin-EDTA when reaching 80%–90% confluence.

### Protein extraction and Western blot analysis

Cultured cells were washed twice with ice-cold PBS and lysed using RIPA buffer (Beyotime) containing 1× protease/phosphatase inhibitor cocktail (Thermo Scientific). The lysates were centrifuged at 12,000 × g for 15 min at 4 °C, and protein concentrations were determined using a BCA assay kit. Equal amounts of protein were separated by 10% SDS-PAGE and transferred onto PVDF membranes (Millipore). After blocking with 5% non-fat milk in TBST for 1 h at room temperature, membranes were incubated overnight at 4 °C with primary antibodies: anti-PDIK1L (Signalway) and anti-GAPDH (Proteintech). Following three washes with TBST, membranes were probed with HRP-conjugated secondary antibodies (Proteintech) for 1 h at room temperature. Protein bands were visualized using ECL Prime substrate and imaged with a ChemiDoc MP imaging system.

### Plasmid transfection

For PDIK1L knockdown or overexpression experiments, C4-2 and PC-3 cells were seeded in 6-well plates at 40%–50% confluence prior to transfection. Transfection complexes were prepared by mixing 2.5 μg of plasmid DNA (pCMV-PDIK1L for overexpression or pLKO.1-shPDIK1L for knockdown) with 5 μL Lipofectamine 3000 reagent (Invitrogen) in Opti-MEM reduced serum medium (Gibco), following the manufacturer’s protocol. Two different shRNA constructs were used: shPDIK1L#1: TGGGCGAATGAAACAACTGAT; shPDIK1L#2: GAAGAACCTGTCAGTGTAAAC. A non-targeting shRNA was used as a control. Following viral transduction, stable polyclonal cell populations were selected with puromycin.

### Cell proliferation assay

PC-3 and C4-2 cells were seeded in 96-well plates at a density of 1,000 cells per well in 100 μL. After 24 h of attachment, cell proliferation was monitored daily for 4 consecutive days using a CCK-8 kit (Beyotime). At each time point, 10 μL CCK-8 reagent was added to each well and incubated at 37 °C for 2 h. Absorbance was measured at 450 nm using a microplate reader. Blank control wells (medium + CCK-8 without cells) were included for background subtraction. Cell viability curves were generated by normalizing daily absorbance values to the Day 0 reading. Each experimental group contained 5 replicate wells.

### Statistical analysis

Statistical analyses were performed using GraphPad Prism 6.0 software and R V.4.2.3. Data from at least three independent biological replicates were normalized to respective controls and expressed as mean ± standard deviation (SD). For comparisons between two groups (e.g., PDIK1L knockdown vs. control, or overexpression vs. empty vector), a two-tailed unpaired Student’s t-test was applied. Comparisons across multiple groups (e.g., time-dependent proliferation assays or multi-dose treatments) were analyzed by one-way ANOVA followed by Tukey’s *post hoc* test for pairwise comparisons. In all analyses, statistical significance was defined as *P* < 0.05 (*), *P < 0.01 (**), *P* < 0.001 (***), and *P* < 0.0001 (****).

## Results

### Metabolic heterogeneity implicated diverse clinical outcomes in prostate cancer microenvironment

**FIGURE 1:**
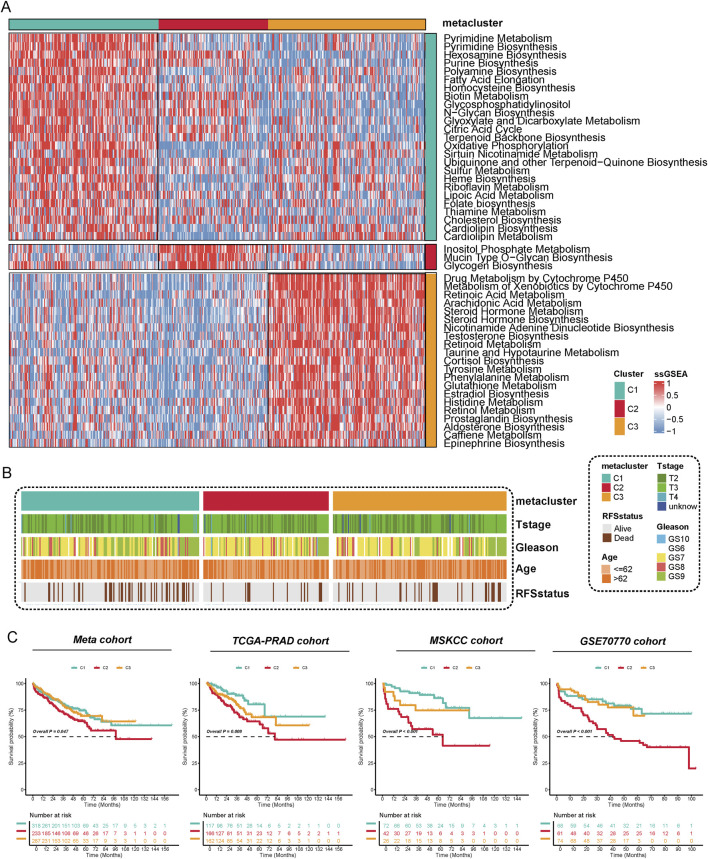
Metabolic Reprogramming Characteristics and Prognostic Significance of Metabolic Subtyping in Prostate Cancer. **(A)** The activity of 53 metabolic pathways was quantified using the ssGSEA method, revealing functional differences among the three subtypes (C1–C3) at the metabolic level. Each row in the figure represents a distinct metabolic pathway, while each column corresponds to a sample. Red indicates upregulation of pathway activity, whereas blue indicates downregulation. The results indicate significant differences among the subtypes in pathways such as purine metabolism, fatty acid elongation, glycogen synthesis, and steroid biosynthesis, suggesting that each subtype has a unique metabolic reprogramming pattern. **(B)** The distribution of different metabolic subtypes was analyzed based on clinicopathological characteristics (T stage, Gleason score, age, and recurrence status). The findings revealed that the C2 subtype was enriched in T3/T4 stages, high Gleason scores, older age groups, and recurrent populations. **(C)** Kaplan-Meier analysis of recurrence-free survival (RFS) for metabolic subtypes was performed in four independent cohorts: Meta, TCGA-PRAD, MSKCC, and GSE70770.

According to cophenetic coefficient and prior articles (Meng et al., 2021), we identified three clusters as the optimized cluster number (Supplementary Figure S1B), and all of the patients were divided into three metabolic populations based on NMF (Supplementary Figure S1C). Metabolic pathway profiling identified three distinct clusters (C1, C2, C3), each enriched with specific pathways (Figure 1A). For instance, Pyrimidine Metabolism and Pyrimidine Biosynthesis were enriched in C1, Glycogen Biosynthesis in C2, while pathways such as Drug Metabolism by Cytochrome P450 were specific to C3. Further analysis revealed that these metabolic clusters are associated with different clinicopathological features in PCa (Figure 1B). We further evaluated the prognostic value of the three subtypes for prostate cancer patients across multiple cohorts. In the Meta cohort, significant RFS were observed among the three subtype groups (*P* = 0.047), with the C2 subtype demonstrating the poorest prognosis. This trend was consistently replicated in independent validation cohorts, including TCGA-PRAD (*P* = 0.008), MSKCC (*P* < 0.001), and GSE70770 (*P* < 0.001), indicating that the C2 subtype is consistently associated with poor survival outcomes, while the C1 subtype exhibited a more favorable survival advantage (Figure 1C). These results support the strong prognostic predictive capability of our constructed molecular classification across multiple independent cohorts.

### Genetic and genomic features of metabolic clusters in prostate cancer

**FIGURE 2:**
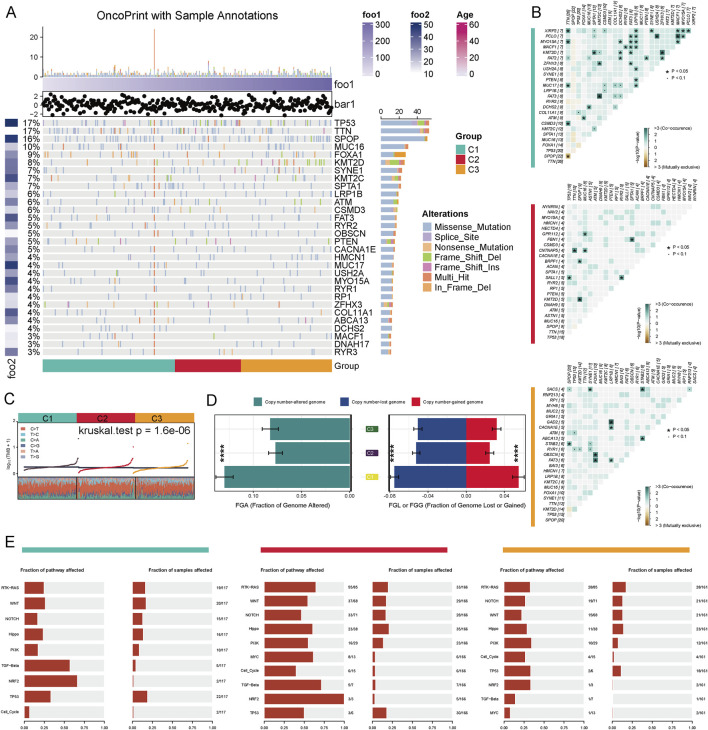
Genomic Alteration Characteristics of Different Metabolic Subtypes in Prostate Cancer. **(A)** The driver gene mutation landscape of three metabolic subtypes (C1–C3) in the TCGA-PRAD cohort is presented. The top 30 genes exhibiting the highest mutation frequencies, along with their respective mutation types, are displayed. These mutation types include missense mutations, splice site mutations, nonsense mutations, frameshift insertions/deletions, and multi-site mutations. **(B)** Co-mutation and mutual exclusivity relationships of driver genes across different subtypes are illustrated. The color intensity in the figure represents -log10 (Fisher’s test P-value), with '+' and '•' indicating P < 0.05 and P < 0.1, respectively. **(C)** The distribution of tumor mutation burden (TMB) across the three subtypes is shown (Kruskal–Wallis test P = 1.6e−06). **(D)** Copy number variation analysis: The left panel displays the fraction of the genome altered (FGA), while the right panel illustrates the proportions of genome loss (FGL) and genome gain (FGG) (***P < 0.001). **(E)** Mutation enrichment analysis of the three subtypes across 10 classic cancer-related signaling pathways, including RTK-RAS, WNT, NOTCH, TP53, and TGF-beta, is provided.

We performed a comprehensive analysis of the genetic features associated with distinct metabolic clusters in prostate cancer. Our analysis of mutation distributions revealed that TP53, TTN, SPOP, and MUC16 were the most frequently mutated genes, with varying mutation rates across different clusters (Figure 2A). To further explore the co-mutation patterns of driver genes across different PCa subtypes, we analyzed the co-occurrence and mutual exclusivity of key gene pairs. The results indicated that in the C1 subtype, TP53 and SPOP exhibited a significant mutually exclusive mutation relationship (*P* < 0.05), while FOXA1 and ARID1A displayed a notable tendency for co-mutation. In the C2 subtype, despite the overall low mutation burden, a co-mutation trend between SPOP and ATM was still observed (*P* < 0.1). In contrast, the C3 subtype exhibited a higher frequency of mutations in driver genes such as TP53, SPOP, and FOXA1, accompanied by significant co-mutation patterns, including the combinations of FOXA1 with MED12 and TP53 with ATM (*P* < 0.05). These findings suggest that the three subtypes exhibit distinct differences in their mutational driving mechanisms, with the C3 subtype demonstrating stronger mutational synergy, indicating a more complex genetic background and tumor evolutionary pathway. (Figure 2B). Using the Kruskal–Wallis’s test, we detected significant variation in TMB among the three clusters (C1, C2, C3) (*P* = 1.6e-06) (Figure 2C). We also evaluated the fraction of FGA and the fraction of FGL/G. Compared to C2 and C3, C1 exhibited a higher FGA, indicating more extensive genomic instability. Similarly, FGL/G analysis showed that C1 had a greater proportion of genomic losses or gains, further highlighting its genomic instability (Figure 2D). We evaluated the enrichment characteristics of gene mutations in typical cancer-related signaling pathways across various subtypes of prostate cancer. The results revealed that the C2 subtype exhibited a significantly higher frequency of pathway alterations, involving multiple driver pathways such as RTK-RAS, WNT, NOTCH, Hippo, PI3K, MYC, TGF-β, NRF2, and TP53, which suggests greater genomic instability in this subtype. Over 50% of the samples were found to simultaneously affect five or more pathways. In contrast, the pathway alterations in the C1 and C3 subtypes were relatively concentrated and less extensive, primarily focusing on the TP53 and TGF-β pathways. This indicates that the C2 subtype may represent a more heterogeneous and aggressive molecular subtype (Figure 2E). These findings underscore the unique genetic and genomic features associated with different metabolic profiles in prostate cancer and suggest potential therapeutic targets based on the integration of metabolic and genetic characteristics.

### Impact of metabolic subtypes on PARP inhibitor sensitivity in prostate cancer

**FIGURE 3:**
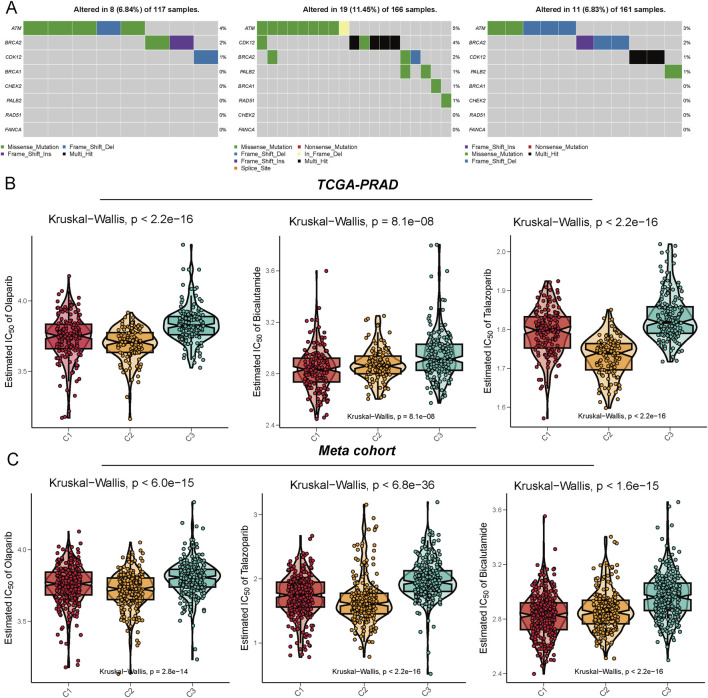
Illustration of the mutation characteristics of the DNA damage repair (DDR) pathway and the analysis of drug sensitivity predictions across different metabolic subtypes. **(A)** This panel displays the mutation profiles of the C1, C2, and C3 subtypes within the TCGA-PRAD cohort, focusing on DDR-related genes such as ATM, BRCA1/2, CHEK2, and PALB2. The mutation types include missense mutations, nonsense mutations, frameshift insertions/deletions, and splice site mutations. **(B,C)** The drug sensitivity prediction results demonstrate the varying responses of the three subtypes to several clinically relevant drugs, specifically Olaparib, Bicalutamide, and Talazoparib. These results were assessed based on the Meta cohort **(B)** and the TCGA-PRAD cohort **(C)**, with the vertical axis representing the predicted IC50 values. Lower IC50 values indicate a higher sensitivity to PARP inhibitors and anti-androgen therapies. Statistical testing was conducted using the Kruskal-Walli’s test.

Genomic analysis of homologous recombination repair (HRR)-related genes across three metabolic subtypes (C1, C2, and C3) revealed distinct mutation patterns in prostate cancer cohorts. C2 subtypes identified a higher alteration frequency of 11.45% (19/166), enriched in nonsense mutations and splice site variants, suggesting compromised DNA repair mechanisms (Figure 3A). Metabolic subtyping stratified tumors into C1, C2 and C3 with C2 exhibiting significantly enhanced sensitivity to PARP inhibitors. In TCGA-PRAD, C2 tumors demonstrated lower IC50 values for olaparib and talazoparib, supported by robust Kruskal–Wallis tests (Figure 3B). Similarly, the meta-cohort highlighted superior olaparib and talazoparib efficacy in C2, alongside pronounced bicalutamide sensitivity (Figure 3C). These results emphasize that metabolic subtyping (C2) correlates with HRR gene alterations and predicts enhanced vulnerability to PARP inhibitors, offering a biomarker-driven framework for targeting DNA repair-defective prostate cancers.

### Different immune landscape and immunotherapy response among the three groups

**FIGURE 4:**
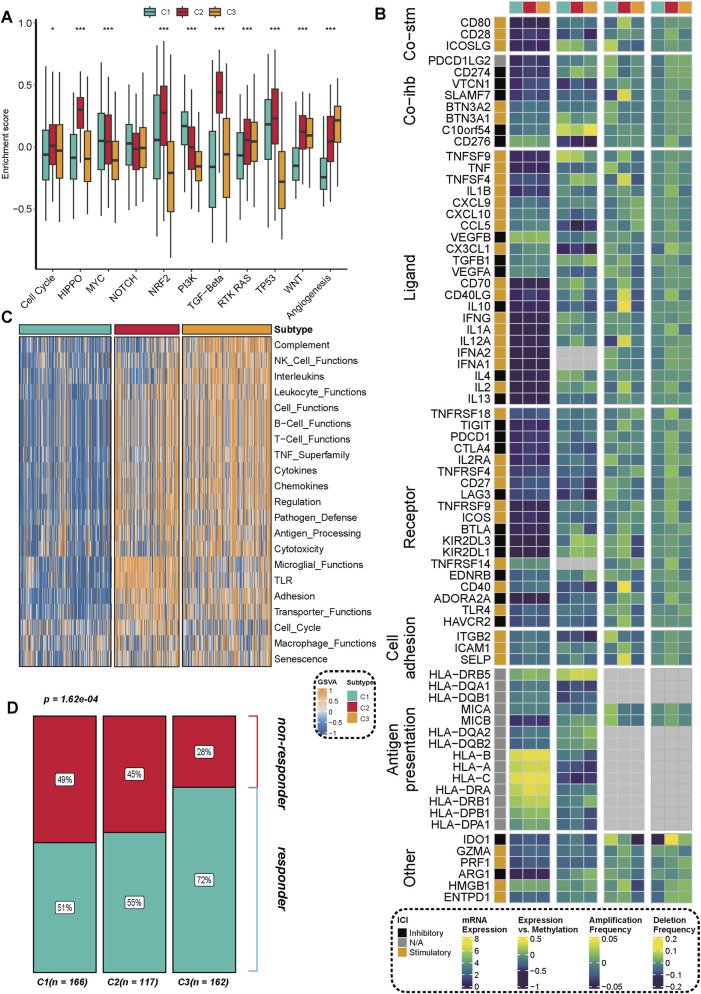
Distribution of immune pathway activation, immune regulatory factor expression, and immunotherapy response characteristics across different subtypes. **(A)** GSVA enrichment analysis revealed significant differences in the activation levels of multiple key immune and signaling pathways among the three subtypes (C1, C2, C3). Statistical significance was assessed using the Kruskal-Walli’s test, with **p < 0.01** and ***p < 0.001**. **(B)** The expression characteristics of key immune regulatory factors, including co-stimulation (Co-stim), co-inhibition (Co-inh), and ligand-receptor (Ligand-Receptor) interactions, are presented, integrating multi-omics information such as mRNA expression, DNA methylation, and copy number amplification/deletion frequency. **(C)** The heatmap illustrates the differential distribution of various immune function modules (e.g., the complement system, NK cell function, chemokines, antigen processing and presentation, etc.) across different subtypes. **(D)** The proportion of immunotherapy responders (Responder), predicted based on the TIDE algorithm, shows significant differences among the different subtypes. The number of samples for each subtype is indicated in parentheses in the figure.

We further analyzed the activities of immune pathways, the expression characteristics of immune regulatory factors, and their potential responses to immunotherapy in three subtypes of prostate cancer. The results of the GSVA analysis revealed significant differences among the subtypes across multiple classic tumor-related immune pathways. The C2 subtype demonstrated notable upregulation in pathways such as Cell Cycle, MYC, TP53, PI3K, and RTK-RAS, indicating a higher level of pathway activation (Figure 4A). In conjunction with previous research (Thorsson et al., 2018), we assessed the expression differences of three categories of immune modulators (co-stimulatory, co-inhibitory, ligand/receptor, etc.) and further integrated data on copy number alterations (SCNA), methylation, and miRNA regulation for expression regulation analysis. Various immune modulators (such as PDCD1LG2, BTN3A1, CXCL10, VEGFA, etc.) exhibited significant differences across the different subtypes, particularly with elevated expression of certain immune suppression-related genes in the C2 subtype. These genes showed a negative correlation with methylation levels, suggesting potential epigenetic regulation (Figure 4B). The C2 and C3 subtypes exhibited significant enhancements across multiple immune function modules, including the complement system, NK cell function, leukocyte function, antigen presentation, chemokines, and cytotoxicity modules. In contrast, the C1 subtype generally demonstrated a trend of suppression, as illustrated in Figure 4C. By further integrating the TIDE immunotherapy prediction score, we found that patients with C2 and C3 subtypes exhibited a higher proportion of responders to immunotherapy across multiple independent cohorts. In contrast, patients with the C1 subtype were predominantly found in the non-responder group (Figure 4D), indicating their reduced potential for responding to immunotherapy.

### Presence of TLS contributed to the high immunotherapy response rate of C2

**FIGURE 5:**
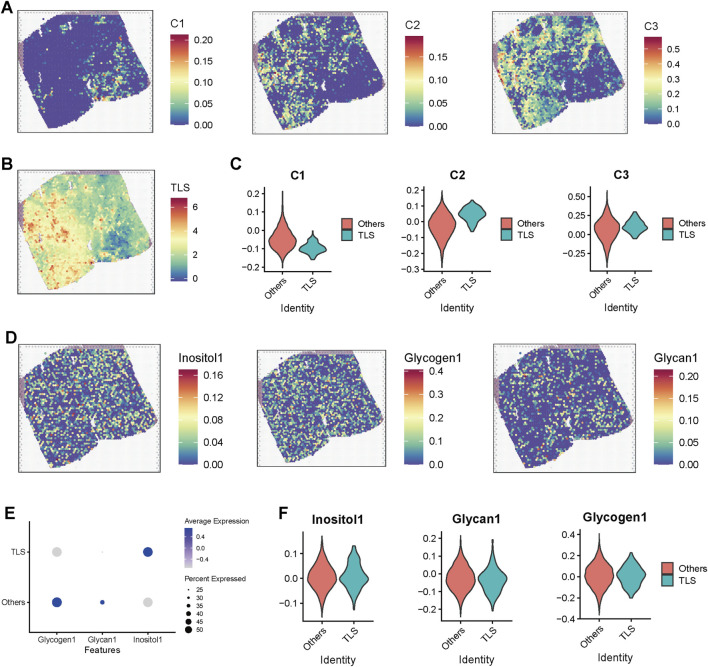
Spatial transcriptomic analysis reveals the association of TLS regions with metabolic pathway activation and C2 subtype enrichment. **(A)** Heatmap displaying scores for modules C1, C2, and C3 in spatial transcriptomic sections. **(B)** Spatial distribution of TLS scores across the sections, with red areas indicating enrichment of TLS structures. **(C)** Violin plot comparing the differences in subtype scores (C1–C3) between TLS and non-TLS regions. **(D)** Spatial distribution maps of inositol metabolism (Inositol1), glycogen metabolism (Glycogen1), and glycosylation (Glycan1) pathways across the sections, demonstrating higher metabolic scores in TLS-enriched regions, which suggests their association with metabolic reprogramming. **(E)** Dot plot illustrating the average expression levels and proportions of three metabolic pathway scores in TLS and non-TLS regions. **(F)** Violin plot further comparing the expression differences of the three metabolic scores between TLS and non-TLS regions.

Our preliminary research found that, despite the high activation of immunosuppressive pathways such as TGF-β and the infiltration of numerous immune cells in C2 subtype prostate cancer samples, these samples still exhibited a favorable response rate to immunotherapy. This seemingly paradoxical phenomenon suggests that specific immune regulatory mechanisms may be at play in this subtype. Further analysis revealed a significant increase in B-cell infiltration in the C2 subtype, indicating a potential close correlation with the formation of tumor-associated TLS. To investigate the association between the functional states of different regions in spatial transcriptomics and the structure of TLS, we evaluated the spatial distribution of three key functional modules (C1–C3) and TLS-related scores in tumor tissue sections (Figures 5A,B). The results indicated that the C2 module score was significantly elevated in TLS-enriched regions, suggesting a close relationship between this functional module and the formation and maintenance of TLS (Figure 5C). To further explore the metabolic characteristics of the TLS region, we introduced the Inositol (inositol metabolism), Glycogen (glycogen metabolism), and Glycan (glycosylation pathway) scores, which were displayed on tissue sections (Figure 5D). The results demonstrated a significant enhancement trend of these three metabolic scores in TLS-enriched regions. Further comparison validated the elevated scores of inositol metabolism in TLS region (Figures 5E,F), suggesting that inositol metabolism pathway may play a crucial role in the TLS microenvironment, potentially involved in maintaining immune cell functions and local immune regulation.

### PDIK1L defines a high-risk C2 subtype with poor survival and enhanced PARP inhibitor sensitivity

**FIGURE 6:**
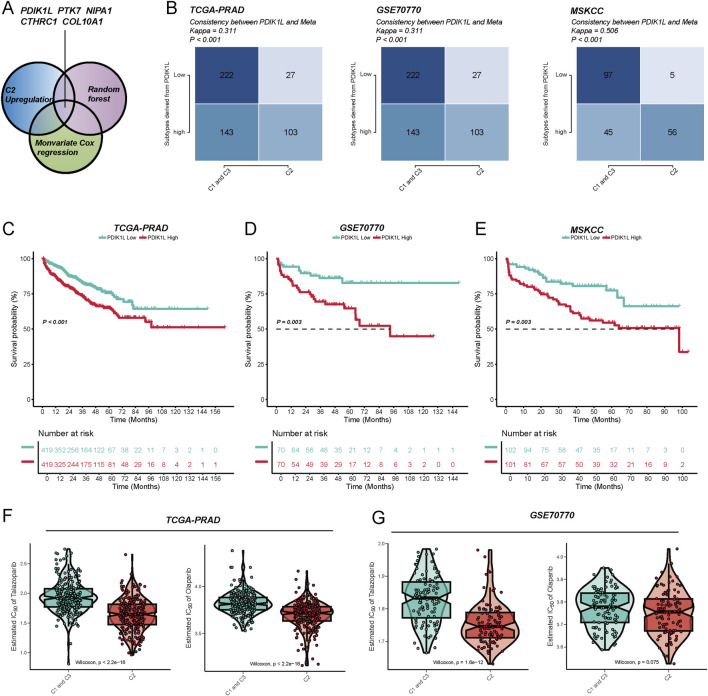
The metabolic score defined by PDK1L can effectively predict patient prognosis and drug sensitivity. **(A)** By utilizing the intersection of upregulated genes in the C2 subtype, univariate Cox regression, and the random forest algorithm, five key metabolism-related genes (PDK1L, PTK7, NIPA1, CTHRC1, COL10A1) were identified to construct the metabolic score model. **(B)** In the TCGA-PRAD, GSE70770, and MSKCC cohorts, the expression levels of PDK1L demonstrated good consistency with the subtype distribution (Meta cluster/C1-C3 grouping), and the Kappa value was significant, indicating that the model exhibits stable typing ability across different cohorts. Kaplan–Meier survival analysis revealed that patients with high PDK1L expression experienced significantly poorer prognoses in the TCGA-PRAD **(C)**, GSE70770 **(D)**, and MSKCC **(E)** cohorts. Drug sensitivity analysis indicated that the low PDK1L expression group displayed higher sensitivity (lower IC50) to drugs such as Tazobactam and Olaparib in the TCGA-PRAD **(F)** and GSE70770 **(G)** cohorts, suggesting that PDK1L may serve as a potential predictive biomarker.

Through integrative analysis, candidate genes were prioritized using a Venn diagram (Figure 6A) approach combining three strategies: (1) upregulated genes in the C2 subtype (Supplementary Table S2), (2) monovariate Cox regression for survival-associated genes (*P* < 0.05) (Supplementary Table S3), and (3) feature selection via random forest algorithm (Supplementary Figures S2A,B). This multi-step screening identified PDIK1L as a key candidate, which was consistently linked to the C2 subtype across three independent cohorts. Patients classified into the C2 subtype exhibited significantly elevated PDIK1L expression (Kappa consistency: 0.311–0.506, all *P* < 0.001) (Figure 6B). Survival analyses revealed that high PDIK1L expression correlated with poor prognosis, as demonstrated by markedly reduced survival probabilities in all cohorts (log-rank *P* = 0.003 and *P* < 0.001) (Figures 6C–E). The high-expression group showed a steep decline in survival over 144 months, with risk tables confirming progressive attrition in this subgroup. Furthermore, PDIK1L-high patients displayed enhanced sensitivity to PARP inhibitors, evidenced by significantly lower estimated IC50 values for Olaparib and Talazoparib (Figures 6F,G). These findings underscore PDIK1L as a prognostic biomarker and a potential predictor of therapeutic response to PARP inhibition in C2-subtype malignancies.

### PDIK1L drives prostate cancer proliferation and modulates PARP inhibitor sensitivity

**FIGURE 7:**
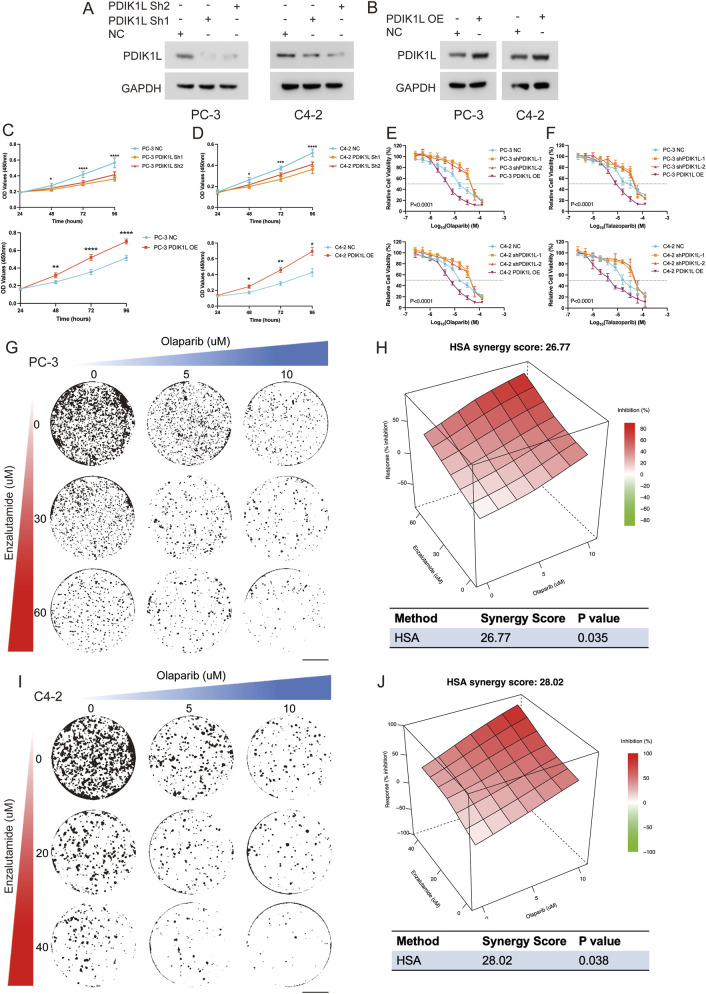
Functional Validation of PDIK1L in Prostate Cancer Proliferation and PARP Inhibitor Response. **(A,B)** Western blot analysis confirming PDIK1L knockdown (Sh1/Sh2) and overexpression (OE) in C4-2 and PC-3 cells. GAPDH was used as loading control. **(C,D)** Cell proliferation curves of C4-2 and PC-3 cells following PDIK1L overexpression or knockdown, measured by CCK-8 assay at indicated time points. **(E,F)** IC50 assays evaluating the effects of Olaparib or talazoparib treatment in PDIK1L-modulated C4-2 and PC-3 cells. **(G–J)** Cell viability assays evaluating the effects of combined Enzalutamide and Olaparib treatment in PDIK1L-modulated C4-2 and PC-3 cells, while synergy score plots based on the HSA model.

Figures 7A,B first validated successful modulation of PDIK1L expression in C4-2 and PC-3 via Western blot. Overexpression (OE) of PDIK1L significantly elevated its protein levels compared to vector controls, while knockdown (Sh1/Sh2) effectively suppressed endogenous PDIK1L expression. Subsequent functional assays (Figures 7C,D) demonstrated that PDIK1L-OE cells exhibited accelerated proliferation, with OD values at 48–96 h markedly higher than controls. Conversely, PDIK1L knockdown suppressed proliferation in both lines. Figures 7E,F further explored therapeutic implications, revealing that PDIK1L-OE cells displayed reduced sensitivity to PARP inhibitors (Olaparib and Talazoparib), with significantly higher IC50 values. In contrast, PDIK1L knockdown sensitized cells to these inhibitors, as shown by sharply decreased relative cell viability.

Enzalutamide is a first-line standard of care for CRPC patients; however, resistance to Enzalutamide remains a major clinical challenge. Emerging evidence indicates that altered glycogen metabolism contributes to Enzalutamide resistance. Our data show that PDIK1L not only promotes prostate cancer proliferation but also enhances glycogen metabolism and improve sensitivity to PARP inhibitors. Given this dual role, we hypothesized that PDIK1L-driven metabolic reprogramming might create a context where PARP inhibitor therapy could be potentiated by co-treatment with Enzalutamide. Strikingly, combination therapy with Enzalutamide and Olaparib synergistically reduced viability in PDIK1L-OE cells, suggesting a strategy to overcome PDIK1L-mediated resistance (HAS Score: 26.77 for PC-3, HAS Score: 28.02 for C4-2; Figures 7G–J). Collectively, these results confirm that PDIK1L drives prostate cancer proliferation and modulates PARP inhibitor efficacy, positioning it as both a therapeutic target and predictive biomarker for combinatorial therapies.

## Discussion

Our study establishes metabolic subtyping as a powerful tool to dissect the interplay between genomic instability and therapeutic vulnerabilities in prostate cancer. The identification of the C2 subtype, characterized by TP53 mutations, elevated genomic instability, and sensitivity to PARP inhibitors, aligns with emerging evidence that metabolic reprogramming creates context-specific dependencies in cancer. Notably, the C2 subtype’s association with HRR defects mirrors clinical observations in HRR-altered tumors treated with PARP inhibitors (Dong et al., 2024). This metabolic-genomic crosstalk is further supported by recent proteogenomic studies revealing distinct molecular subtypes in high-risk prostate cancer, where metabolic pathways like branched-chain amino acid metabolism drive tumor progression (Dong et al., 2024; Ou et al., 2025).

The interplay between metabolic heterogeneity and immune evasion mechanisms in prostate cancer represents a critical axis for therapeutic intervention. Our spatial transcriptomic analysis revealed that TLS-enriched regions in C2 tumors exhibit elevated inositol and glycogen metabolism, suggesting a metabolic-immune symbiosis that sustains immune cell activity. This finding aligns with emerging evidence that metabolic byproducts (e.g., lactate, inositol derivatives) can modulate immune checkpoint expression and T-cell exhaustion (Llibre et al., 2025; Burke et al., 2023). Notably, the C2 subtype’s paradoxical association with both immunosuppressive pathways (e.g., TGF-β) and favorable immunotherapy responses may stem from TLS-mediated antigen presentation, which counterbalances local immunosuppression. Recent studies in renal cell carcinoma similarly demonstrate that TLS-associated metabolic niches enhance PD-1 inhibitor efficacy by fostering cytotoxic T-cell infiltration (Xu et al., 2020; Cabrita et al., 2020). These observations underscore the need to evaluate metabolic-immune crosstalk when designing combination therapies, particularly for C2 tumors where metabolic inhibitors (e.g., glycogen synthase kinase inhibitors) could potentiate immunotherapy by remodeling the immune landscape.

The prioritization of PDIK1L as a C2-specific biomarker underscores its dual role in tumor proliferation and therapy resistance. Mechanistically, PDIK1L may stabilize oncogenic signaling by modulating DNA repair or metabolic adaptations, akin to OTUD6A, a deubiquitinase recently shown to stabilize c-Myc and promote metabolic remodeling in prostate cancer (Peng et al., 2022). Our finding that PDIK1L overexpression confers PARP inhibitor resistance parallels studies demonstrating that MYC-driven metabolic rewiring compromises therapeutic efficacy (Peng et al., 2022; Schaub-Clerigue et al., 2025). Conversely, PDIK1L knockdown sensitizes cells to PARP inhibition, suggesting its potential as a therapeutic target. This dual functionality is reminiscent of PGC1α, which exerts non-cell autonomous tumor suppression by regulating secreted factors like spermidine synthase, highlighting the complexity of metabolic regulation in tumor-microenvironment interactions (Schaub-Clerigue et al., 2025).

Beyond its role in PARPi resistance, PDIK1L may serve as a master regulator linking metabolic reprogramming to DNA repair dysregulation. Mechanistically, PDIK1L’s interaction with nucleotide biosynthesis pathways could deplete dNTP pools, exacerbating replication stress and genomic instability in C2 tumors—a phenomenon observed in BRCA1-deficient cancers treated with PARPi (Mateo et al., 2015). This dual capacity to drive proliferation while impairing DNA repair mirrors the oncogenic role of MYC, which similarly coordinates anabolic metabolism and replication stress (Meena et al., 2024). Furthermore, PDIK1L’s association with TP53 mutations suggests a feedforward loop wherein metabolic dysregulation stabilizes mutant p53, perpetuating genomic chaos (Liu et al., 2024). Targeting this axis via PDIK1L inhibition could simultaneously curb proliferation and restore HRR competency, offering a synthetic lethality approach for C2 tumors. Future studies should delineate whether PDIK1L directly modulates HRR components or acts through intermediary metabolites, which would refine therapeutic strategies.

The clinical implications of our work are twofold. First, metabolic subtyping offers a stratification framework to identify patients most likely to benefit from PARP inhibitors, particularly in HRR-deficient C2 tumors. This approach complements recent advances in prostate-specific membrane antigen (PSMA)-targeted imaging, which improves diagnostic precision but lacks predictive value for therapy response (Su et al., 2025). Second, the synergy between enzalutamide and olaparib in overcoming PDIK1L-mediated resistance aligns with trials exploring androgen receptor and PARP inhibitor combinations, emphasizing the need for biomarker-driven combinatorial strategies (Dalla Volta et al., 2025).

While this study establishes a framework for metabolic subtyping, several limitations must be acknowledged. First, the reliance on retrospective cohorts may introduce selection bias; prospective validation in diverse populations is essential. Second, the functional role of PDIK1L in modulating DNA repair requires deeper mechanistic investigation, particularly its interaction with HRR components like BRCA1/2. Third, the clinical applicability of our findings depends on scalable biomarker assays, such as ctDNA-based monitoring of metabolic signatures.

## Conclusion

In conclusion, our findings bridge metabolic diversity with genomic instability in prostate cancer, positioning PDIK1L as a pivotal node for therapeutic intervention. By integrating multi-omics insights and functional validation, this work advances precision oncology paradigms, offering a roadmap for targeting metabolic vulnerabilities in high-risk disease.

## Data Availability

The datasets presented in this study can be found in online repositories. The names of the repository/repositories and accession number(s) can be found in the article/Supplementary Material.
