# Supplementary figures and images for: Metabolic subtyping reveals PDIK1L as a dual-functional regulator of progression and PARP inhibitor sensitivity in prostate cancer
Source: Front Cell Dev Biol. 2025 Nov 4;13:1674844. doi: 10.3389/fcell.2025.1674844 (PMC12623405; doi:10.3389/fcell.2025.1674844)

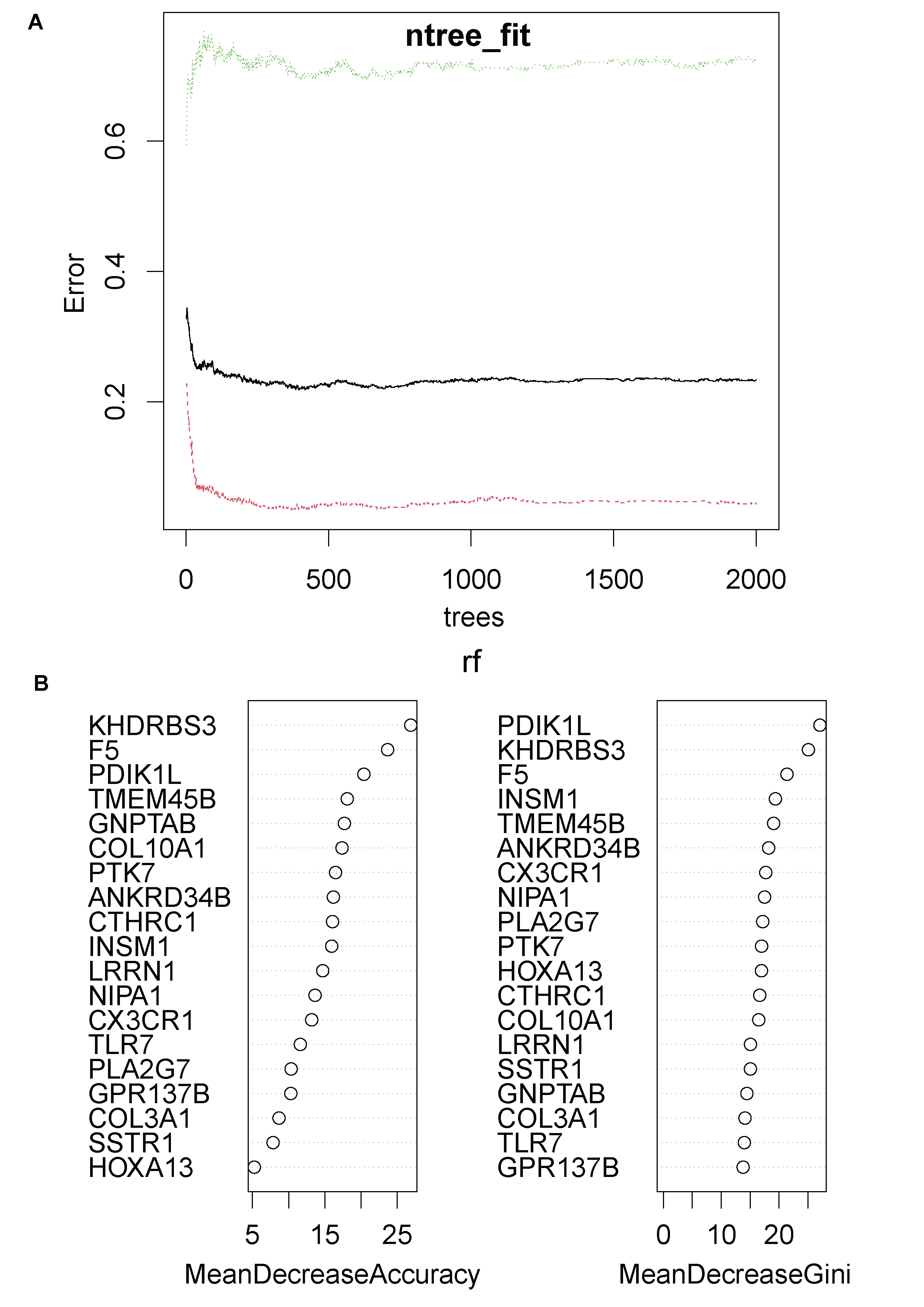

Supplement: Supplementary file 3 [file Image2.tif]

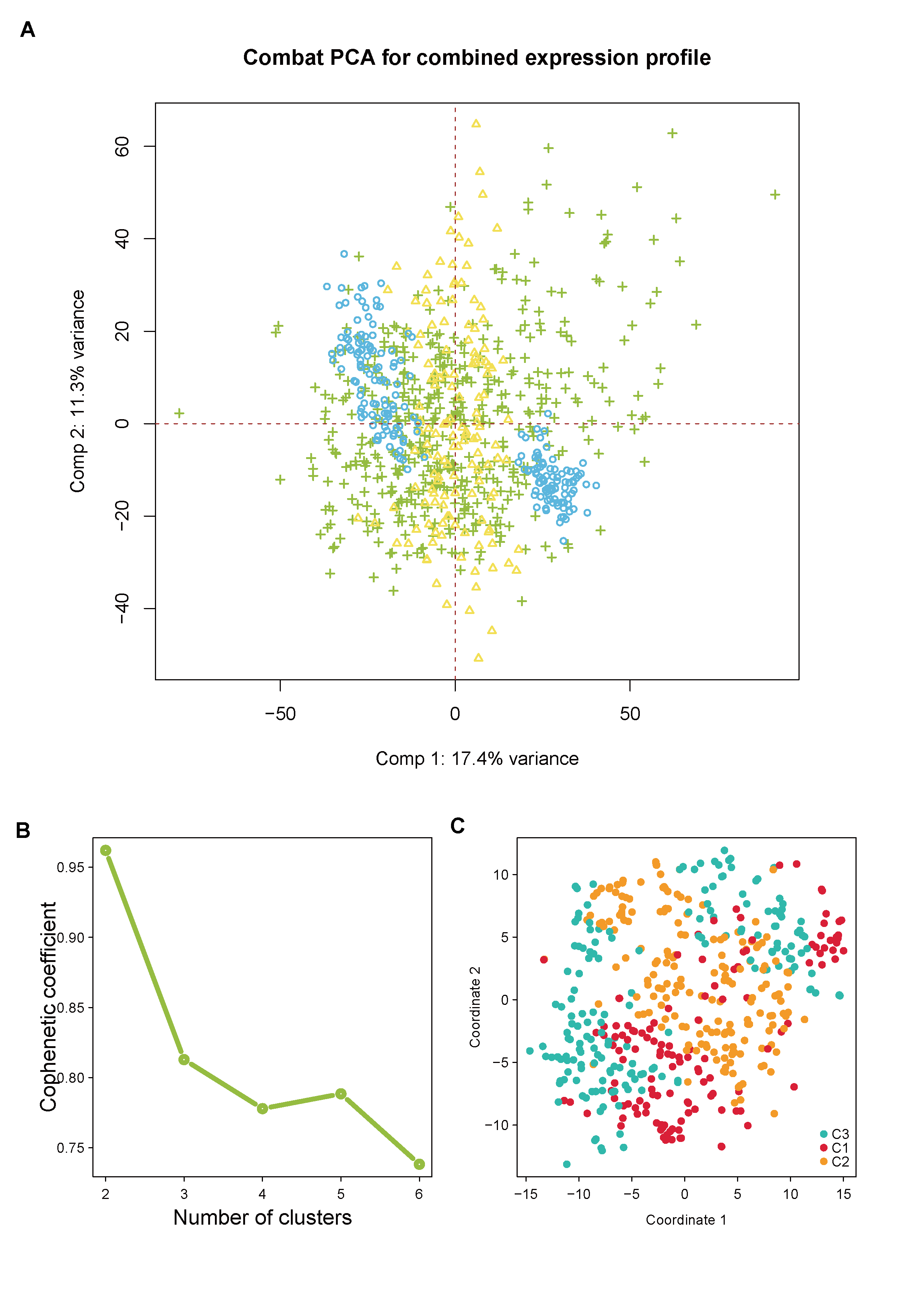

Supplement: Supplementary file 4 [file Image1.tif]
